# The association between paternal diabetes mellitus and successful pregnancy—Examined in a nationwide population undergoing reproductive treatment

**DOI:** 10.1111/andr.13702

**Published:** 2024-07-30

**Authors:** Anne‐Sofie Sønnichsen‐Dreehsen, Jens Fedder, Mette Wod, Caroline Thingholm Thorarinsson, Bente Mertz Nørgård

**Affiliations:** ^1^ Centre of Andrology & Fertility Clinic Odense University Hospital & University of Southern Denmark Odense Denmark; ^2^ Center for Clinical Epidemiology Odense University Hospital Odense Denmark; ^3^ Research Unit of Clinical Epidemiology Department of Clinical Research University of Southern Denmark Odense Denmark

**Keywords:** clinical epidemiology, diabetes mellitus, paternal, pregnancy, type 1 diabetes: type 2 diabetes

## Abstract

**Background:**

About 15% of all pregnancies end in pregnancy loss. As most studies have focused on maternal factors little is known regarding the influence of paternal factors on the chance of successful pregnancy.

**Objectives:**

This cohort study aims to assess the chance of biochemical pregnancy, clinical pregnancy, and live‐born children in couples where the male partner has diabetes mellitus (DM).

**Materials and methods:**

We performed a nationwide cohort study. Couples undergoing assisted reproductive technology treatment from 2006 to 2019 were included. The exposed cohorts comprised embryo transfers in couples with paternal type 1 DM (T1DM), type 2 DM (T2DM), or mixed type DM (TMDM). The unexposed cohort included embryo transfers in couples without paternal DM.

**Results:**

A total of 101,875 embryo transfers were included. Of these, 503 males had T1DM, 225 males had T2DM, 263 males had TMDM, and 100,884 did not have DM. For paternal T1DM, the adjusted OR for achieving a biochemical pregnancy, clinical pregnancy, and live‐born child were 0.97 (95% CI 0.77–1.23), 1.08 (95% CI 0.65–1.79), and 0.75 (95% CI 0.49–1.14), respectively. For paternal T2DM, the adjusted OR for achieving a biochemical pregnancy, clinical pregnancy, and live‐born child were 0.80 (95% CI 0.56;1.16), 0.67 (95% CI 0.32–1.41), and 1.03 (95% CI 0.48–2.20), respectively. For the paternal TMDM, the adjusted OR for achieving a biochemical pregnancy, clinical pregnancy and livebirth were 0.95 (95% CI 0.67–1.33), 1.31 (95% CI 0.56–2.92), and 1.19 (95% CI 0.59–2.38), respectively.

**Conclusion:**

Paternal DM was not associated with a statistically significant decreased chance of biochemical pregnancy, clinical pregnancy, or live birth

## 1 INTRODUCTION

Approximately 15% of all pregnancies end in early pregnancy loss or clinical miscarriage^1^, ^2^ causing tremendous psychological pain for the couples affected.^3^, ^4^ Most studies regarding unsuccessful pregnancies have focused on maternal factors and found that increased female age, chromosomal abnormalities, anatomic abnormalities, infections, and autoimmune disorders all increase the risk of pregnancy loss.^5^ Reversible maternal risk factors have also been linked to pregnancy loss including smoking, alcohol, obesity, and caffeine consumption.^6^, ^7^

Little is known about how paternal factors might influence a couple's chance of successful pregnancy. A few studies have suggested that advanced paternal age,^8^ low levels of education,^9^ obesity,^10^ and smoking^11^ affect male fertility, pregnancy outcomes, and childhood health and development negatively. Varicocele,^12^ metabolic syndrome^13^ as well as diseases in the respiratory system, nervous system, and endocrine system^14^ in males might also be related to adverse pregnancy outcomes, but the evidence is still low.^7^ These findings indicate that it is important to research the possible association between paternal health and the chances of live births.

Diabetes mellitus (DM) is a common chronic disease with increasing incidence and prevalence. It is a condition with elevated levels of blood glucose due to impaired cell uptake of carbohydrates.^7^, ^15^ Broadly, DM is split into type 1 and type 2. Type 2 (T2DM) is the most common type of DM and is characterized by insulin resistance.^16^ Type 1 DM (T1DM) is an autoimmune condition with destruction of beta cells resulting in progressive insulin deficiency.^16^ Compared with T2DM, T1DM is frequently diagnosed at a younger age.^17^ DM represents serious health issues because of how it affects many functions and organs of the body including the reproductive system.^18^ Maternal DM has been found to impair female fertility^19^ and increase the rates of adverse pregnancy outcomes such as hypertension, pre‐eclampsia, abnormal fetal growth, preterm birth, stillbirth, and caesarean section.^20^, ^21^, ^22^, ^23^ Male fertility might be impaired by DM due to disruption of spermatogenesis leading to decreased sperm motility and increased DNA fragmentation.^18^, ^24^, ^25^, ^26^ Retrograde ejaculation due to diabetic neuropathy has also been found to cause impaired fertility among males with T1DM or T2DM.^27^, ^28^, ^29^ Furthermore, males with DM and retrograde ejaculation are more likely to exhibit erectile dysfunction compared with males without DM.^29^ The results of these studies indicate that paternal DM might influence a couple's ability to conceive a child but the evidence is still low. Further research is needed to improve the chances of successful pregnancies.

In this cohort study, we aimed to examine whether paternal DM was associated with a decreased chance of conceiving a child or a live birth compared with nonpaternal DM. We estimated the chance of (1) a biochemical pregnancy, (2) a clinical pregnancy, and (3) a live‐born child.

## 2 MATERIALS AND METHODS

### 2.1 Setting and design

We used Danish national health registries to conduct a nationwide cohort study covering a study period of 2006 through 2019. The study focuses on couples undergoing assisted reproductive technology (ART) treatment—specifically in vitro fertilization technique (IVF) and intracytoplasmic sperm injection (ICSI). Data on early pregnancy outcomes were available in this population that is, a pregnancy test two weeks after the embryo transfer, an ultrasonic scan after another three weeks, and a complete follow‐up on all pregnancies that result in a live‐born child.

### 2.2 Data sources

Data for this study were derived from several Danish health registries. The Civil Registration System provides information on the entire Danish population including the civil registration number, death, and migration. It is used across all Danish health registries and can be used to link information from population registers on an individual level.^30^ The Danish ART registry includes data on all ART procedures in Denmark including causes of infertility, and the achievement of a biochemical pregnancy, clinical pregnancy, and live birth. All single women and infertile couples are offered up to three fresh fertility treatment attempts in Denmark. However, the woman must be younger than 41 years of age when being referred to ART treatment. The ART registry includes mandatory registration data from all treatment cycles in public and private clinics, and it was established on 1 January 1994.^31^, ^32^, ^33^ We used the most recent version of the ART registry containing data on all ART treatments since 2006. The older version containing information on the ART registries from 1994 to 2005 was not included since it lacked information on transfers using frozen embryo replacements (FER). The Medical Birth Register contains data on all births in Denmark since 1973 and includes detailed information on the mother, father, birth, and child.^34^, ^35^ The National Patient Registry gives information on hospitalizations of all Danish patients including diagnoses based on the International Classification of Diseases—ICD‐8 before 1994 and ICD‐10 from 1994 and onwards.^36^ These data were used to identify males with any type of DM.

### 2.3 The study population

The study population included all couples registered in the ART registry with an embryo transfer from 1 January 2006 up until 1 January 2019. We included those who received IVF‐ or ICSI treatments with either fresh cycle embryo transfers or FER embryo transfers. Couples using donor eggs and/or donor spermatozoa as well as embryo transfers resulting in multiple pregnancies were excluded. We also excluded couples receiving other kinds of ART treatment such as insemination and hormone therapy since these patients were not followed as closely as couples receiving IVF‐ or ICSI treatment. Another reason for not including couples undergoing insemination is that our dataset and all our analyses were built upon the embryo transfer date, which we did not have access to among couples undergoing insemination.

### 2.4 Exposed and unexposed

The study population was divided into two main cohorts—one cohort contained male partners with DM before the egg retrieval (exposed cohort) and the other cohort contained male partners without any DM before the egg retrieval (unexposed cohort). The exposed cohort was identified using ICD‐codes (primary and secondary discharge diagnoses) (ICD‐8: 249* and 250*, ICD‐10: DE10* and DE11*). Until 1987 T1DM and T2DM were classified with the same ICD‐code in the National Patient Registry making it impossible to distinguish between the two.^37^, ^38^ All male partners in the exposed cohort were only included if they had at least two DM diagnoses after 1987 and at least one of them had to be registered before the egg retrieval.

The exposed cohort was further divided into three—one cohort included all couples where the male partners had T1DM, the second cohort included the couples where the male partners had T2DM, and the third cohort included male partners with diagnoses of both T1DM and T2DM. This cohort will be referred to as mixed‐type DM (TMDM). T1DM was defined as having at least two diagnoses for T1DM (ICD‐8: 249 or ICD‐10: DE10) and no diagnoses for T2DM. T2DM was defined as having at least two diagnoses for T2DM (ICD‐8:250 or ICD‐10: DE11) and no diagnoses for T1DM. TMDM was defined as having at least one diagnosis for T1DM and one for T2DM.

The unexposed cohort included embryo transfers where the male partners had no records of a DM diagnosis before the egg retrieval. If the male partners included in the study were given a DM diagnosis between two egg retrievals, they would be included in the unexposed cohort at the first egg retrieval and in one of the exposed cohorts depending on the type of DM at the second egg retrieval. Given that some patients with diabetes receive care exclusively from their general practitioners, we conducted sensitivity analyses on antidiabetic treatments. We allocated exposure groups according to antidiabetic medications instead of diabetic diagnostic codes: (1) insulin (ATC: A10A) without other antidiabetic treatment (ATC: A10B) and (2) other antidiabetic medication with or without insulin.

### 2.5 Statistical analysis

We estimated the OR for the chance of pregnancies in couples with male partners having DM relative to male partners without DM. A multilevel logistic regression with a 95% confidence interval (CI) was used to estimate our outcome. Multilevel logistic regression takes into account that couples could have multiple embryo transfers during the study period. The regression model was adjusted for male age (continuous), female age (continuous), female smoking status (yes/no), female alcohol intake (yes/no), female body mass index (BMI; underweight [<18.5], normal [18.5–24.99], overweight [24.99–29.99], obese [>30]), earlier pregnancy loss (yes/no), calendar year of ART treatment (2006–2009, 2010−2014, 2015−2019), cause of infertility treatment(male, female, both), and the type of infertility treatment (IVF, ICSI, IVF+FER, ICSI+FER). Alcohol intake “yes” is defined as ≥1 drink during a week.

The effect of other covariates was also tested. These included male Charlson Comorbidity Index (0/≥1), female Charlson comorbidity index (0/≥1), and parity (0/+1). The Charlson comorbidity index was constructed based on data from the Danish National Patient Registry. However, none of these variables changed the conclusion and were not included in the final model. We did not test the effect of male smoking status (yes/no) and male alcohol intake (yes/no) due to large proportions of missing data. All statistical analyses were performed using Stata version 18.0 (StataCorp.).

### 2.6 Ethics, approvals, and data availability

No approval by an ethics committee in Denmark is required for register‐based studies before they can be carried out. The project was approved under the research notification in the Region of Southern Denmark/the Danish Data Protection Agency (journal number 22/13116). The authors of this paper have no special access privileges to the data used in this study. Other researchers can apply for access to data from the Research Service at the Danish Health Data Authority.

### 2.7 Patient and public involvement

Our patient representatives who are members of the research council for the Center for Clinical Epidemiology were part of the initial process of this project.

## 3 RESULTS

Table 1 shows descriptive characteristics of the study population. The study included 101,875 couples with a known male partner undergoing ART treatment in the form of either IVF or ICSI from 2006 to 2019. Among these, 503 men had T1DM, 225 had T2DM, and 263 had TMDM. The cohorts were homogenous on parameters such as calendar year of ART treatment, cause of infertility, and type of ART treatment. Both men and women were older in the cohort with T2DM compared with the other cohorts. Of the women in the T2DM cohort, 24.9% were obese compared with 6.8% in the T1DM, 11.8% in the TMDM, and 9.8% in the unexposed cohort. Couples in the T1DM cohort had experienced earlier pregnancy loss in 69.6% of cases compared with 26.2% in the T2DM cohort, 26.2% in the TMDM, and 31.5% in the unexposed cohort.

**TABLE 1:** Characteristics of all men and women included in the 4 cohorts: Diabetes mellitus (DM) type 1(T1DM), DM type 2 (T2DM), DM mixed type (TMDM), unexposed in the study period of January 2006–September 2019.

	Exposed cohort (embryo transfers in women whose partners have T1DM) *N* = 503	Exposed cohort (embryo transfers in women whose partners have T2DM) *N* = 225	Exposed cohort (embryo transfers in women whose partners have TMDM) *N* = 263	Unexposed cohort (embryo transfers in women whose partners do not have DM) *N* = 100,884
**Male characteristics**				
Median age at egg retrieval (25 percentile–75 percentile)	36 (32–40)	41 (37–46)	39 (34–45)	35 (32–40)
Smoking status				
Smoker, *N* (%)	76 (15.1)	37 (16.4)	44 (16.7)	13,041 (12.9)
Nonsmoker, *N* (%)	332 (66.0)	150 (66.7)	151 (57.4)	69,607 (69.0)
Missing, *N* (%)	95 (18.9)	38 (16.9)	68 (25.9)	18,236 (18.1)
Alcohol intake				
Yes, *N* (%)	270 (53.7)	78 (34.7)	106 (40.3)	53,144 (52.7)
No, *N* (%)	135 (26.8)	105 (46.7)	77 (29.3)	27,429 (27.2)
Missing, *N* (%)	98 (19.5)	42 (18.7)	80 (30.4)	20,331 (20.1)
Charlson comorbidity index				
0, *N* (%)	313 (62.3)	149 (66.2)	167 (63.5)	94,878 (94.0)
≥1, *N* (%)	190 (37.8)	76 (33.8)	96 (36.5)	6006 (6.0)
**Female characteristics**				
Median age at egg retrieval (25 percentile–75 percentile)	34 (30–37)	37 (33–40)	34 (30–39)	34 (30–37)
Smoking status				
Smoker, *N* (%)	59 (11.7)	25 (11.1)	28 (10.6)	7572 (7.5)
Nonsmoker, *N* (%)	388 (77.2)	184 (81.8)	194 (73.8)	83,509 (82.8)
Missing, *N* (%)	56 (11.1)	16 (7.1)	41 (15.6)	9803 (9.7)
Alcohol intake				
Yes, *N* (%)	221 (43.9)	56 (24.9)	91 (34.6)	39,206 (38.9)
No, *N* (%)	216 (43.0)	149 (66.2)	117 (44.5)	49,781 (49.3)
Missing, *N* (%)	66 (13.1)	20 (8.9)	55 (20.9)	11,897 (11.8)
Charlson comorbidity index				
0, *N* (%)	456 (90.7)	202 (89.8)	237 (90.1)	93,716 (92.9)
>1, *N* (%)	47 (9.3)	23 (10.2)	26 (9.9)	7168 (7.1)
Body mass index (BMI)				
<18.5 (underweight), *N* (%)	14 (2.8)	0 (0)	8 (3.0)	3125 (3.1)
18.5–24.99 (normal), *N* (%)	272 (54.1)	76 (33.8)	109 (41.4)	54,728 (54.3)
25.00–29.99 (overweight), *N* (%)	123 (24.4)	59 (26.2)	72 (27.4)	23,531 (23.3)
>30.00 (obese), *N* (%)	34 (6.8)	56 (24.9)	31 (11.8)	9871 (9.8)
Missing	60 (11.9)	34 (15.1)	43 (16.4)	9629 (9.5)
Earlier pregnancy loss				
Yes, *N* (%)	350 (69.6)	59 (26.2)	69 (26.2)	31,769 (31.5)
No, *N* (%)	153 (30.4)	166 (73.8)	194 (73.8)	69,115 (68.5)
Parity				
0, *N* (%)	432 (85.9)	210 (93.3)	232 (88.2)	87,523 (86.8)
1+, *N* (%)	71 (14.1)	15 (6.7)	31 (11.8)	13,361 (13.2)
Calendar year of ART				
2006–2009, *N* (%)	147 (29.2)	62 (27.6)	92 (35.0)	28,068 (27.8)
2010–2014, *N* (%)	175 (34.8)	95 (42.2)	111 (42.2)	39,623 (39.3)
2015–2019, *N* (%)	181 (36.0)	68 (30.2)	60 (22.8)	33,193 (32.9)
Cause of infertility				
Male, *N* (%)	194 (38.6)	64 (28.4)	98 (37.2)	29,021 (28.8)
Female, *N* (%)	141 (28.0)	69 (30.7)	83 (31.6)	33,625 (33.3)
Both, *N* (%)	144 (28.6)	84 (37.3)	66 (25.1)	34,419 (34.1)
Missing, *N* (%)	24 (4.8)	8 (3.6)	16 (6.1)	3819 (3.8)
Type of ART treatment				
IVF	188 (37.4)	82 (36.4)	109 (41.4)	43,346 (43.0)
ICSI	236 (46.9)	119 (53.0)	129 (49.1)	44,303 (43.9)
IVF+FER	43 (8.5)	12 (5.3)	12 (4.6)	7353 (7.3)
ICSI+FER	36 (7.2)	12 (5.3)	13 (4.9)	5882 (5.8)

The crude and adjusted ORs for biochemical pregnancy, clinical pregnancy, and live birth for paternal T1DM are shown in Table 2. The adjusted OR to achieve a biochemical pregnancy was 0.97 (95% CI 0.77–1.23), the adjusted OR to achieve a clinical pregnancy was 1.08 (95% CI 0.65–1.79), and 0.75 (95% CI 0.49–1.14) for a live‐born child. The adjusted OR to achieve a biochemical pregnancy and a clinical pregnancy for paternal T2DM was 0.80 (95% CI 0.56–1.16) and 0.67 (95% CI 0.32–1.41), respectively. The adjusted OR to achieve a live‐born child was 1.03 (95% CI 0.48–2.20; Table 3). The adjusted OR to achieve a biochemical pregnancy and clinical pregnancy for paternal TMDM was 0.95 (95% CI of 0.67–1.33) and 1.31 (95% CI 0.58–2.92), respectively. The adjusted OR to achieve a live‐born child was 1.19 (95% CI 0.59–2.38; Table 4). In the sensitivity analyses, using antidiabetic medications as exposure assessment instead of diabetic diagnostic codes, the conclusions of the study did not change (data not shown).

**TABLE 2:** The chance of biochemical pregnancy, a clinical pregnancy, and live birth after assisted reproductive technology (ART) treatments in a study population of 101,875 embryo transfers with 503 belonging to the exposed cohort of women whose partner has diabetes mellitus (DM) type 1 (T1DM), and 100,884 belonging to the unexposed cohort.

	Exposed cohort (embryo transfers in women whose partners have T1DM)	Unexposed cohort (embryo transfers in women whose partners do not have DM)	Crude OR (95% CI)	Adjusted OR (95% CI)^a^
Biochemical pregnancy (hCG)^b^				
Yes, *N* (%)	201 (40.0)	38,677 (38.3)	1.07 (0.86–1.34)	0.97 (0.77–1.23)
No, *N* (%)	302 (60.0)	62,207 (61.7)		
Clinical pregnancy (ultrasound)^c^				
Yes, *N* (%)	167 (83.1)	33,084 (85.5)	0.82 (0.54–1.25)	1.08 (0.65–1.79)
No, *N* (%)	34 (16.9)	5593 (14.5)		
Live birth^d^				
Yes, *N* (%)	125 (74.9)	26,371 (79.7)	0.76 (0.51–1.13)	0.75 (0.49–1.14)
No, *N* (%)	42 (25.1)	6713 (20.3)		

^a^ Adjusted for male age, female age, female smoking status, female alcohol intake, female body mass index (BMI), earlier pregnancy loss, calendar year of ART, cause of infertility treatment, and type of ART treatment.

^b^ Number of embryo transfers in the exposed cohort: (503). Number of embryo transfers in the un*exposed* cohort: (100,884).

^c^ Number of biochemical pregnancies in the exposed cohort: (201). Number of biochemical pregnancies in the unexposed cohort: (38,677).

^d^ Number of clinical pregnancies in the exposed cohort: (167). Number of clinical pregnancies in the unexposed cohort: (33,084).

**TABLE 3:** The chance of biochemical pregnancy, a clinical pregnancy, and live birth after assisted reproductive technology (ART) treatments in a study population of 101,875 embryo transfers with 225 belonging to the exposed cohort of women whose partner has diabetes mellitus (DM) type 2 (T2DM), and 100,884 belonging to the unexposed cohort.

	Exposed cohort (embryo transfers in women whose partners have T2DM)	Unexposed cohort (embryo transfers in women whose partners do not have DM)	Crude OR (95% CI)	Adjusted OR (95% CI)^a^
Biochemical pregnancy (hCG)^b^				
Yes, *N* (%)	65 (28.9)	38,677 (38.3)	0.66 (0.46–0.93)	0.80 (0.56–1.16)
No, *N* (%)	160 (71.1)	62,207 (61.7)		
Clinical pregnancy (ultrasound)^c^				
Yes, *N* (%)	52 (80.0)	33,084 (85.5)	0.66 (0.33 –1.31)	0.67 (0.32–1.41)
No, *N* (%)	13 (20.0)	5593 (14.5)		
Live birth^d^				
Yes, *N* (%)	39 (75.00)	26,371 (79.7)	0.72 (0.36–1.45)	1.03 (0.48–2.20)
No, *N* (%)	13 (25.00)	6713 (20.3)		

^a^ Adjusted for male age, female age, female smoking status, female alcohol intake, female body mass index (BMI), earlier pregnancy loss, calendar year of ART, cause of infertility treatment, and type of ART treatment.

^b^ Number of embryo transfers in the exposed cohort: (225). Number of embryo transfers in the unexposed cohort: (100,884).

^c^ Number of biochemical pregnancies in the exposed cohort: (65). Number of biochemical pregnancies in the unexposed cohort: (38,677).

^d^ Number of clinical pregnancies in the exposed cohort: (52). Number of clinical pregnancies in the unexposed cohort: (33,084).

**TABLE 4:** The chance of biochemical pregnancy, a clinical pregnancy, and live birth after assisted‐reproductive technology (ART) in a study population of 101,875 embryo transfers with 263 belonging to the exposed cohort of women whose partner has diabetes mellitus (DM) mixed type (TMDM) and 100,884 belonging to the unexposed cohort.

	Exposed cohort (embryo transfers in women whose partners have TMDM)	Unexposed cohort (embryo transfers in women whose partners do not have DM)	Crude OR (95% CI)	Adjusted OR (95% CI)^a^
Biochemical pregnancy (hCG)^b^				
Yes, *N* (%)	93 (35.4)	38,677 (38.3)	0.85 (0.63–1.16)	0.95 (0.67–1.33)
No, *N* (%)	170 (64.6)	62,207 (61.7)		
Clinical pregnancy (ultrasound)^c^				
Yes, *N* (%)	82 (88.2)	33,084 (85.5)	1.26 (0.63–2.52)	1.31 (0.58–2.92)
No, *N* (%)	11 (11.8)	5593 (14.5)		
Live birth^d^				
Yes, *N* (%)	69 (84.2)	26,371 (79.7)	1.36 (0.71–2.58)	1.19 (0.59–2.38)
No, *N* (%)	13 (15.8)	6713 (20.3)		

^a^ Adjusted for male age, female age, female smoking status, female alcohol intake, female body mass index (BMI), earlier pregnancy loss, calendar year of ART, cause of infertility treatment, and type of ART treatment.

^b^ Number of embryo transfers in the exposed cohort: (263). Number of embryo transfers in the unexposed cohort: (100,884).

^c^ Number of biochemical pregnancies in the exposed cohort: (93). Number of biochemical pregnancies in the unexposed cohort: (38,677).

^d^ Number of clinical pregnancies in the exposed cohort: (82). Number of clinical pregnancies in the unexposed cohort: (33,084).

## 4 DISCUSSION

To our knowledge, this is the largest study to investigate the association between paternal DM and the chance of successful pregnancy. Reassuringly, none of our results were statistically significant meaning that paternal DM did not seem to influence a couple's chance of a successful pregnancy. This was seen in all types of DM included in our study (T1DM, T2DM, TMDM).

Only a few minor studies with a limited ability to adjust for confounders have previously focused on investigating whether paternal DM affects a couple's chance of pregnancy.^39^, ^40^ A small study by Raju et al.^39^ compared 35 noninsulin‐dependent DM males with 123 controls. Males with noninsulin‐dependent DM had decreased sperm motility and increased DNA fragmentation. The fertilization rate was not influenced by an increased sperm DNA fragmentation but a negative correlation between DNA fragmentation and blastocyst formation was found. Couples with diabetic male partners had significantly lower pregnancy rates (28.57%) compared with couples with nondiabetic male partners (46.34%; *p* < 0.001).^39^ Couples with diabetic male partners had significantly higher miscarriage rates (50.0%) compared with couples with nondiabetic male partners (24.56%; *p* < 0.001).^39^ In a retrospective chart review, Mulholland et al.^40^ found 80 couples with a male DM partner of which 18 went through ART treatment. The fertilization rates were comparable with couples with non‐DM male partners (IVF: 68% in the DM vs. 70% in the non‐DM, ICSI: 62% in the DM vs. 71% in the non‐DM).^40^ The pregnancy rate of fresh cycle embryo transfers led to one pregnancy (5%) which was a lower rate than expected (28%). The pregnancy rate of embryo transfers using FER (29%) was similar to the expected (21.3%).^40^ Our results did not reflect those found by Raju et al.^39^ and Mulholland et al.^40^ We were able to include a larger study population and divided the exposed cohort into three types of DM. This gave us the ability to investigate whether the chance of successful pregnancy depended on the type of DM, which our results reassuringly did not show. We did not include fertilization ratios in our study, as we wanted to focus on the pregnancy rates and live‐born rates. Raju et al.^39^ and Mulholland et al.^40^ did not look at the rate of live‐born children as was done in our study. Our data which included the whole sequence of reproductive events gave us the ability to follow up on every pregnancy and, thus, get a more detailed insight into how paternal DM influences the chance of a successful pregnancy.

Other researchers have focused on other aspects related to DM and male infertility. In a study by Barkabi‐Zanjani et al.,^41^ it was indicated that oxidative stress might influence the signaling pathways in spermatogenesis leading to a reduced chance of impregnating a woman. A study by Condorelli et al.^42^ found that the pathophysiology behind male infertility and T1DM was different from male infertility and T2DM. They did not investigate whether DM decreased the chance of successful pregnancy. Still, their results suggested that the type of DM might influence the chances of getting a child differently. A review by Lotti et al.^43^ investigated the existing evidence on the possible effects of DM on sperm quality and fertility outcomes. The study found it difficult to reach any robust conclusions on how DM might influence semen parameters and sexual hormones in men. Lotti et al.^43^ suggested that further studies would be needed before any conclusions could be made. The aim of this study was not to investigate the semen parameters in diabetic and nondiabetic males, but our results showed that paternal DM did not influence the chance of a successful pregnancy.

Our study has several strengths including very strict inclusion criteria for the patients in the exposed cohort to ensure an accurate assessment of exposure in the study. To decrease the risk of misclassification of the exposure assessment^44^, ^45^ men were only included if they had at least two DM diagnoses after 1987 and at least one of them had to be registered before the egg retrieval. Some patients with DM do not receive pharmacological treatment but use diet and exercise alone as treatment even though they have been registered with a DM diagnosis.^17^, ^46^ Therefore, we believe that the use of ICD‐codes for DM is the most valid method for the identification of patients with DM. Based on the ICD codes, we were able to stratify DM into three categories (T1DM, T2DM, and TMDM). It would be difficult to stratify DM into categories by using antidiabetic treatment since some antidiabetics such as insulin can be used to treat both T1DM and T2DM.^17^ Distinguishing between the types of DM is a strength of our study as the biological mechanism of T1DM and T2DM is different.^16^, ^17^ Previous studies have demonstrated that antidiabetic treatment may impact offspring outcomes.^47^, ^48^ Nonetheless, sensitivity analyses conducted using antidiabetic treatment data instead of ICD codes did not alter the results.

Another strength is that the study population was identified using data from the Danish ART registry as it is based on valid and complete data. This also enabled us to investigate the chance of early pregnancy as it is mandatory to report all treatment cycles from both public and private clinics.^31^, ^32^, ^33^ An advantage of the ART registry is that it provides the ability to look at the exact timing of important pregnancy events (biochemical pregnancy, clinical pregnancy, and live‐born child). Around 11% of all Danish children are expected to be born as a result of ART treatment each year, and IVF accounts for 4.5% of all live births in Europe.^49^, ^50^ This made it possible to include a large study population by using the ART registry.

Data were gathered independently of the hypotheses investigated and exposure measurement reducing the risk of information bias. As the registers included in this study have existed for many years it was possible to include a large study population providing us with solid statistical power. Danish health registries are also known for their high data quality and completeness making it possible to study long‐term health outcomes and minimize the risk of loss to follow‐up.^47^

A limitation of register‐based studies is that they often lack information on lifestyle factors and clinical parameters such as severity and dysregulation of DM. It is a limitation that we did not have information on HbA1c values in our dataset, as they could have provided information on diabetes control among the males in the exposed cohorts. It would be interesting to examine how the difference in diabetes control among men with DM could influence the chance of successful pregnancy in future studies.

Another limitation is the risk of missing data. We adjusted for smoking and alcohol in the females but not in the males as the quantity of missing data was much lower in the females. The evidence of an association between smoking and alcohol intake in males and reduced male fertility is however small.^51^ We had no data on ethnicity, male BMI, or socioeconomic status, so the impact of these factors cannot be measured. In a nonrandomized study like this, unknown confounders can never be ruled out, and it is possible that an unknown risk factor or lifestyle factor in the males or females could have influenced our results. Still, even in a large study like this, we were challenged by the statistical power. It is a limitation that our study population might not be representative of the background population since couples undergoing ART treatment have been found to be highly educated, healthier, and live longer than the background population.^52^

## 5 CONCLUSION

This study found that paternal DM did not decrease a couple's chances of achieving a pregnancy or a live‐born child compared with couples without paternal DM. These are reassuring results but need to be confirmed in other studies including data on male lifestyle factors such as smoking and alcohol and clinical parameters such as severity and dysregulation of DM before any firm conclusions can be made.

## AUTHOR CONTRIBUTIONS

All authors contributed to the conception and design, data gathering or analysis, and interpretation of data. All authors were part of writing the article or revised it critically for important intellectual content, gave final approval of the version to be published, and agreed to be responsible for all aspects of the work.

## CONFLICT OF INTEREST STATEMENT

The authors declare no conflict of interest.

## Data Availability

The research data are not publicly available.
